# Insight into the Intermolecular Recognition Mechanism between Keap1 and IKKβ Combining Homology Modelling, Protein-Protein Docking, Molecular Dynamics Simulations and Virtual Alanine Mutation

**DOI:** 10.1371/journal.pone.0075076

**Published:** 2013-09-16

**Authors:** Zheng-Yu Jiang, Hong-Xi Chu, Mei-Yang Xi, Ting-Ting Yang, Jian-Min Jia, Jing-Jie Huang, Xiao-Ke Guo, Xiao-Jin Zhang, Qi-Dong You, Hao-Peng Sun

**Affiliations:** 1 State Key Laboratory of Natural Medicines, China Pharmaceutical University, Nanjing, China; 2 Jiang Su Key Laboratory of Drug Design and Optimization, China Pharmaceutical University, Nanjing, China; 3 Department of Medicinal Chemistry, School of Pharmacy, China Pharmaceutical University, Nanjing, China; 4 Department of Organic Chemistry, School of Science, China Pharmaceutical University, Nanjing, China; MRC National Institute for Medical Research, United Kingdom

## Abstract

Degradation of certain proteins through the ubiquitin-proteasome pathway is a common strategy taken by the key modulators responsible for stress responses. Kelch-like ECH-associated protein-1(Keap1), a substrate adaptor component of the Cullin3 (Cul3)-based ubiquitin E3 ligase complex, mediates the ubiquitination of two key modulators, NF-E2-related factor 2 (Nrf2) and IκB kinase β (IKKβ), which are involved in the redox control of gene transcription. However, compared to the Keap1-Nrf2 protein-protein interaction (PPI), the intermolecular recognition mechanism of Keap1 and IKKβ has been poorly investigated. In order to explore the binding pattern between Keap1 and IKKβ, the PPI model of Keap1 and IKKβ was investigated. The structure of human IKKβ was constructed by means of the homology modeling method and using reported crystal structure of *Xenopus laevis* IKKβ as the template. A protein-protein docking method was applied to develop the Keap1-IKKβ complex model. After the refinement and visual analysis of docked proteins, the chosen pose was further optimized through molecular dynamics simulations. The resulting structure was utilized to conduct the virtual alanine mutation for the exploration of hot-spots significant for the intermolecular interaction. Overall, our results provided structural insights into the PPI model of Keap1-IKKβ and suggest that the substrate specificity of Keap1 depend on the interaction with the key tyrosines, namely Tyr525, Tyr574 and Tyr334. The study presented in the current project may be useful to design molecules that selectively modulate Keap1. The selective recognition mechanism of Keap1 with IKKβ or Nrf2 will be helpful to further know the crosstalk between NF-κB and Nrf2 signaling.

## Introduction

**Figure 1:**
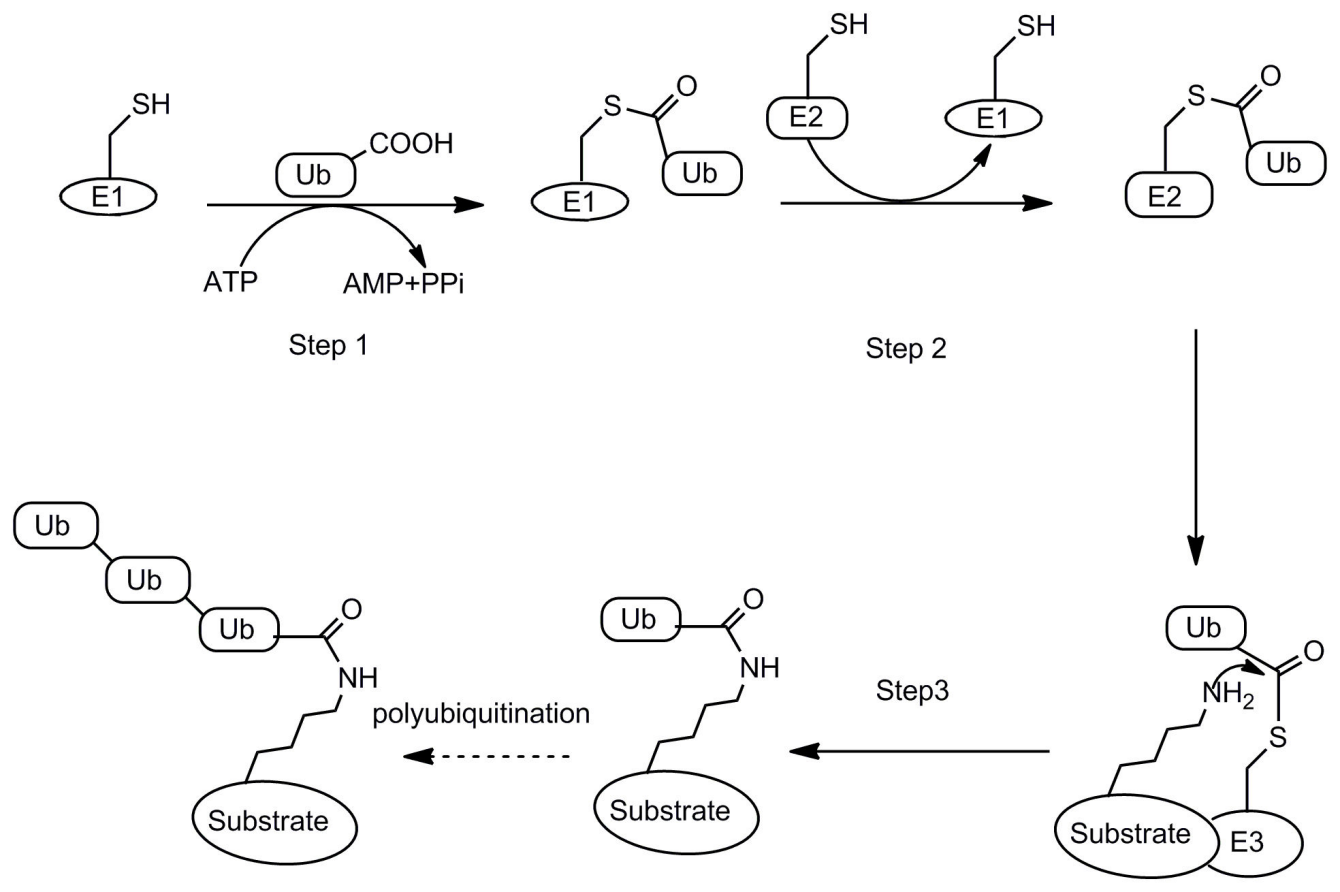
Ubiquitination of a protein substrate through E1→E2→E3. Step1: E1 activates Ub C terminus to form a thioester intermediate by coupling ATP hydrolysis; Step2: the activated Ub is transferred to E2 also in the thioester form; Steps3: the E3 ligase catalyzes the transfer of one Ub molecule at a time or a Ub chain to a protein target.

Ubiquitination is one of the most important posttranslation modifications in eukaryotes [1]. Though ubiquitination has been confirmed to be involved in a wide range of cellular processes, it is mostly known as a proteasomal degradation signal. Ubiquitination of a protein target is tightly regulated through a cascade of enzyme activities (E1→E2→E3) to link the C-terminal Gly residue of ubiquitin (Ub) to the Lys side chain of the target protein through an isopeptide bond [2,3](as shown in Figure 1). In the initial step, E1 activates the Ub C terminus to form a thioester intermediate by coupling ATP hydrolysis [4]. Then the activated Ub is transferred to E2 also in the thioester form. The E3 ligase catalyzes the transfer of one Ub molecule at a time or a Ub chain to a protein target [1,5]. In order to ensure the accuracy and efficiency of ubiquitination, the enzymatic machinery is composed of two E1 enzymes, 30–40 E2 enzymes, and several hundred E3 ligases [1]. Among them, the E2 enzyme is closely related with the linkage type of Ub chain, while the E3 ligase is the main determinant of the substrate specificity [6].

**Figure 2:**
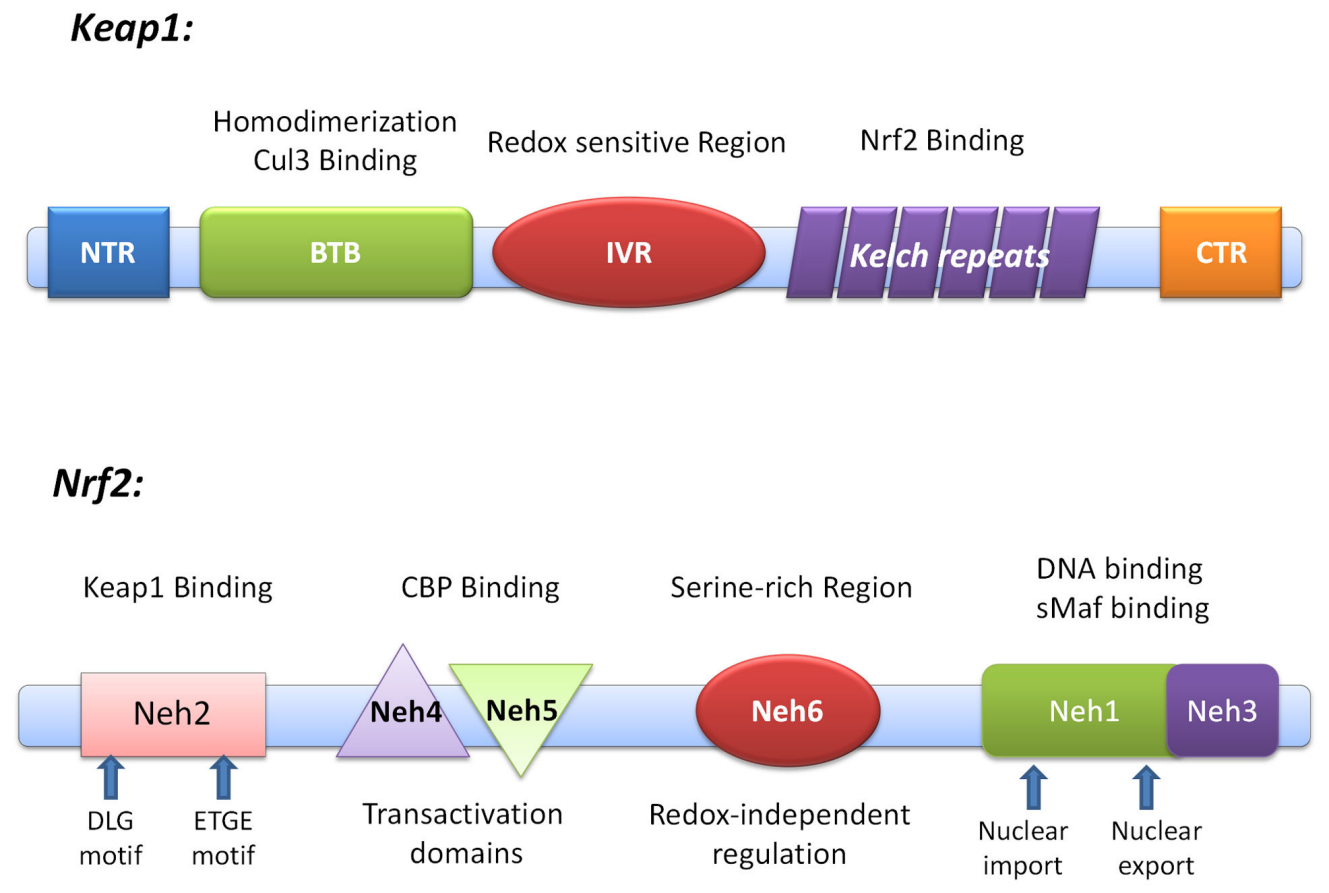
Domain structure of Nrf2 and Keap1. Keap1 possesses four characteristic domains: the Broad complex, Tramtrack, and Bric-a-Brac (BTB); the intervening region (IVR); the double glycine repeat or Kelch repeat (DGR); and the C-terminal region (CTR). The DGR and CTR domains, also known as DC domain, mediate interaction with Neh2 domain of Nrf2. Nrf2 can be divided into six highly homologous regions (Neh1 to Neh6 domains) through comparing the human and chicken Nrf2 amino acid sequences. Neh2 is responsible for interacting with Keap1.

Kelch-like ECH-associated protein-1(Keap1), a Bric-a-Brac (BTB) protein, is a substrate adaptor component of the Cullin3 (Cul3)-based ubiquitin E3 ligase complex which is in charge of recognizing ubiquitination substrates through protein-protein interaction (PPI). There are four characteristic domains in Keap1 (Figure 2): the Broad complex, Tramtrack, and Bric-a-Brac (BTB); the intervening region (IVR); the double glycine repeat (DGR) or the Kelch domain; and the C-terminal region (CTR). Many BTB proteins, including Keap1, have been found to serve as the substrate-specific adaptors for Cul3 ubiquitin ligase [7,8]. Keap1 also possesses multiple reactive cysteines whose reactive thiols are excellent targets of electrophiles. Thus, it is a sensor to monitor the redox homeostasis. The transcription factor NF-E2-related factor 2 (Nrf2), a key inducer of many cytoprotective genes in response to oxidative and electrophilic stresses, is the most well-known substrate of Keap1-Cul3 E3 ligase [9-11]. The DGR and CTR domain, also known as DC domain, mediate the interaction with Neh2 domain of Nrf2.

Besides Nrf2, the DC domain of Keap1 has been reported to bind other proteins, including DJ-1, sequestosome 1, p62, IκB kinase β (IKKβ), Hsp90, p65, Bcl-2/Bcl-xL [12-21]. Among these, IKKβ is the most attractive binding partner [20]. IKKβ functions as a key controller in the canonical nuclear factor kappa enhancer binding protein (NF-κB) cascade. A variety of pro-inflammatory stimuli can activate IKKβ, which then phosphorylate IκB, leading to ubiquitination and subsequent degradation of IκB through the 26S proteasomal pathway. The removal of IκB results in the nuclear translocation of NF-κB, which binds to specific genes [22]. Besides, IKKβ has shown the NF-κB-independent tumorigenicity through phosphorylation-mediated inhibition of tumor suppressors [23].

Nrf2 and NF-κB is the core of redox control of gene transcription [24]. Redox sensitive transcription factors play an important role in regulating several pathways that lead to carcinogenesis and cell survival [20,24]. Keap1 down-regulates the NF-κB signaling pathway by way of functioning as an IKKβ E3 ligase [15]. Induction of Keap1 expression will kill two birds (IKKβ and Nrf2) with one stone, and inactivate two pathways (Keap1-Nrf2-ARE and IKKβ-NF-κB) simultaneously. A large number of pathological stimuli, such as cigarette smoke [25], lipopolysaccharide (LPS) [26,27], oxidized low-density lipoprotein [28], and reactive oxygen species [29], activate both NF-κB signaling and Nrf2-ARE pathway. Nevertheless, NF-κB and Nrf2 plays contrary role in the pathological processes of inflammation and cancer. A variety of anti-inflammatory or anti-cancerogenic phytochemicals suppresses NF-κB signaling and activates Nrf2-ARE pathway as well [30,31]. Although there are emerging evidences supporting a functional interplay between Nrf2 and NF-κB pathways, the regulatory mechanisms which integrate these two functionally opposing pathways and finally determine the transcriptional outputs are still obscure.

Considering the core role of IKKβ and Nrf2 in NF-κB signaling and Nrf2-ARE pathway, the selective recognition mechanism of Keap1 with IKKβ or Nrf2 is vital to the crosstalk between NF-κB and Nrf2 signaling. However, compared to Keap1-Nrf2 system, the intermolecular recognition mechanism of Keap1 and IKKβ has been poorly investigated. In this paper, the structure of human IKKβ modeled from the crystal structure of *Xenopus laevis* IKKβ [32] was firstly reported to investigate the recognition mechanism between Keap1 and IKKβ. Protein-protein docking method was used to construct the IKKβ-Keap1 complex. Molecular dynamics (MD) simulation of Keap1 DC domain for 100 ns indicated that the induced fit interaction may exist between Keap1 and the substrate. MD simulation of IKKβ-Keap1 complex revealed that three tyrosines, namely Tyr525, Tyr574 and Tyr334, are the key point to selectively bind IKKβ. The results from virtual alanine mutation scanning also gave the consistent results. As arginines (such as Arg380, Arg415 and Arg483) were reported crucial for the binding of Nrf2 to Keap1, our results here revealed that IKKβ and Nrf2 are recognized by Keap1 through different molecular mechanism. Considering the mentioned arginines are close to the tyrosines in the same binding site of Keap1, a competitive binding mechanism between IKKβ and Nrf2 may exist, providing the regulatory strategy between the Keap1-Nrf2-ARE and IKKβ-NF-κB pathway crosstalk and the cellular redox homeostasis. Meanwhile, targeting these residues can be helpful for the generation of selective modulators of Keap1.

## Methods

### General procedure

All structures obtained from protein data bank (PDB) were corrected using clean protein tool in Discovery Studio (DS) 3.0 package (Accelrys Inc., San Diego, CA). All calculations were conducted using Dawning TC2600 cluster. Except for otherwise mentioned, other parameters were set as default.

### Homology Modeling of human IKKβ

The sequence of human IKKβ protein was obtained from Swiss-Prot protein database (ID: O14920). The sequence similarity of *Homo sapiens* IKKβ against the PDB sequences was analyzed by the NCBI BLAST server (http://www.ncbi.nlm.nih.gov/blast/). The *Xenopus laevis* IKKβ (PDB code: 3QA8) which was identified as the most homologous sequence from PDB (Identities = 76%, Positives = 86%, File S1) was chosen as the only template structure. Automated sequence alignment and analysis of template and target was carried out through the Align Sequence to Templates protocol in DS 3.0. MODELLER [33], inbuilt in the DS package, was used to automodel the human IKKβ protein sequence. Five models were produced and the value of the Modeler objective function and the ‘discrete optimized potential energy’ (DOPE) score were calculated to evaluate the model. The best model was used in the following procedure.

### Protein-Protein Docking

The crystal structure of Keap1 DC domain was downloaded from PDB (PDB code: 2FLU with high resolution of 1.50 Å). ZDOCK [34], a rigid-body protein-protein docking algorithm based on the Fast Fourier Transform Correlation technique, was used to construct the Keap1-IKKβ complex. Angular step size for rotational sampling of ligand orientations was set to 6°, which can perform finer conformational sampling and typically result in more accurate predictions [35]. In order to filter the docked poses, three important arginines (Arg380, Arg415 and Arg483, which are crucial for the recognition of Nrf2) of Keap1 and the known binding motif of IKKβ (NQE^36^TGE ^39^N) were chosen as the residues including in the binding site [15]. Top 2000 poses were retained for evaluation using ZRANK [36] scoring function. The remaining poses were then processed by the clustering method.

### Refinement of Docked Proteins

RDOCK, an algorithm for refinement of docked complexes using the CHARMm-based minimization, was used to optimize and re-rank the docking poses to pick out near-native structures [37]. The docked poses have been typed with the CHARMm Polar H force field in advance. The parameters were set as default.

### Molecular Dynamics Simulation

The preparation of all molecular files and MD simulations were conducted using the GROMACS package, version 4.5.3. Unless otherwise noted, the all-atom OPLS force field were assigned to all molecules and the simple point charge (SPC) water model was applied to solvate the molecules. Counterions were added to the solvent to keep the system neutral. All bond lengths were constrained using the LINCS method [38], allowing a 2 fs time step. Long-range electrostatic interactions were calculated with the smooth Particle Mesh Ewald (PME) method [39,40]. The neighbor list was updated every five simulation steps (10 fs). The cut-off at 1 nm was applied to van der Waals and Coulombic interactions. Steepest descent minimization was performed until the maximum force < 1000.0 kJ/mol/nm followed by conjugate gradient minimization until the Polak-Ribiere Conjugate Gradients converged to the machine precision (1849 steps for Keap1 and 2972 steps for the complex). Before the MD simulations, the system was equilibrated using position-restrained (PR) MD as follows: i) 1ns of isochoric-isothermal (NVT) equilibration at 300K with V-rescale [41] utilized to control temperature; ii) further equilibration under an isothermal-isobaric (NPT) ensemble was performed for 1ns at the same temperature and 1 bar of pressure. V-rescale was utilized to control temperature and Parrinello-Rahman barostat [42,43] was utilized to control pressure.

Upon completion of the two equilibration phases, the well-equilibrated system at the desired temperature and pressure was used to run the production MD without position restraints. Other parameters were the same as the NPT ensemble in the equilibration phase. The coordinates were saved every 10 ps. The Keap1 DC domain was simulated for 100 ns and the Keap1-IKKβ complex system was simulated for 50 ns. The trjconv tool within the GROMACS was used to strip out coordinates and correct for periodicity. The production MD without position restraints of Keap1 DC domain and Keap1-IKKβ were repeated for three times. The Gromos method [44] within GROMACS (g_cluster) was employed to perform a cluster analysis. RMSD cut-off for two structures to be neighbor was set to 0.1 nm for the Keap DC domain system and 0.2 nm for Keap1-IKKβ complex system. The central structure of the most dominant cluster for each of the simulations was saved for further analysis.

### Virtual Alanine Mutation

The Calculate Mutation Energy (Binding) protocol in DS 3.0 was used to evaluate the effect of single-point mutations on the binding affinity of molecular partners in the protein-protein complex. The energetic effect of each mutation on the binding affinity is calculated as the difference between the binding free energy in the mutated structures and the wild type protein. All interaction energy terms are calculated by CHARMm using a Generalized Born implicit solvent model and contain empirically scaled contributions of van der Waals and electrostatic interactions and a non-polar solvation energy term. The input structure of Keap1-IKKβ complex was obtained from the cluster analysis of the MD simulation trajectory. In order to compare the difference of key residues, the crystal structure of Keap1-Nrf2 (PDB code: 2FLU) was used as the input structure to conduct the virtual alanine mutation. In order to avoid the unreasonable clash, the preliminary minimization was carried out for both cases. The residues within 3.5Å around ETGE motif in Keap1 were selected for calculation. The Energy Terms Scaling Factors were set as default.

## Results

### Homology Modeling *Homo sapiens* IKKβ

**Figure 3:**
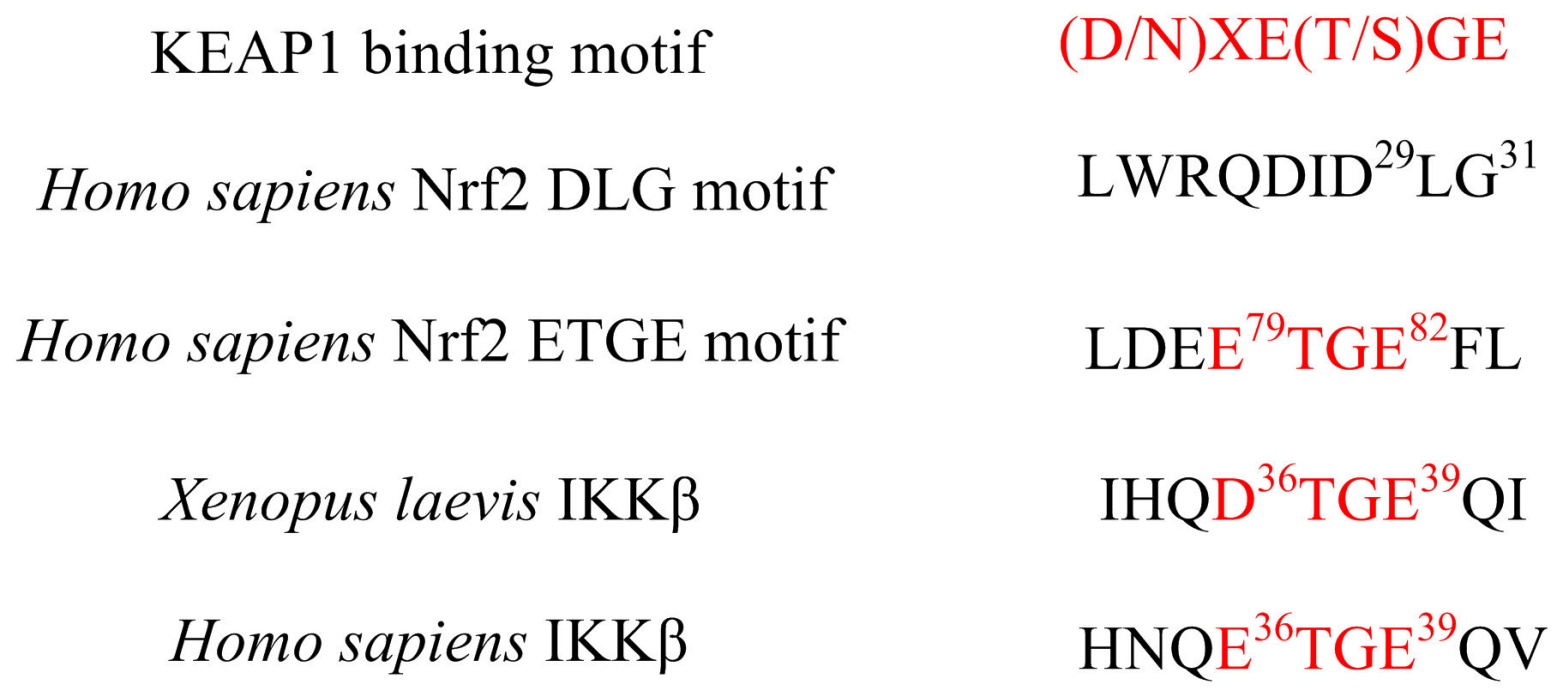
Sequence comparison of Keap1 binding motif. The Asn34 and Gln35 are located before ETGE in human IKKβ, while these two residues change to the Asp77 and Glu78 in Nrf2.

**Figure 4:**
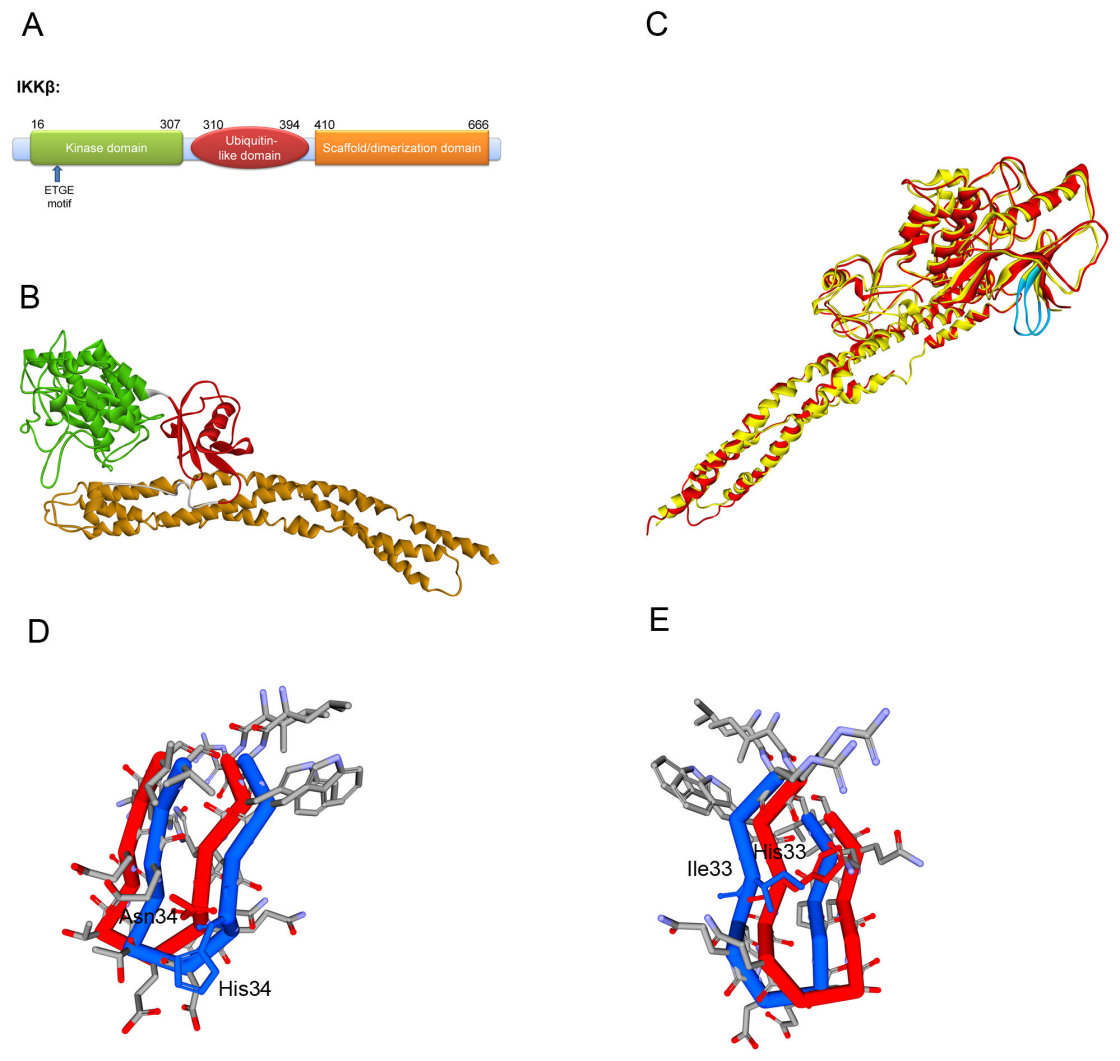
Homology Modeling of *Homo sapiens* IKKβ. (A) Domain structure of IKKβ. IKKβ can be divided into three function domains: a kinase domain (KD), an ubiquitin-like domain (ULD) and an elongated, α-helical scaffold/dimerization domain (SDD) colored as green, red and yellow. (B) Ribbon diagram of *Homo sapiens* IKKβ from Homology Modeling. KD (green), ULD (red) and SDD (yellow) are labeled. (C) Superimpose of *Homo sapiens* IKKβ and *Xenopus laevis* IKKβ. The Keap1 binding motif is colored as azure. In general, the structure of *Homo sapiens* IKKβ (yellow) and *Xenopus laevis* IKKβ (red) are quite similar. Both of the Keap1 binding motifs (blue) form the β-turn structure. (D) & (E) Structure difference of Keap1 binding motif between *Homo sapiens* IKKβ (red) and *Xenopus laevis* IKKβ (blue). The most different residues were labeled in the picture.

In general, the average homology model accuracy is a function of the template-target sequence similarity [45]. The only known crystal structure IKKβ is obtained from *Xenopus laevis*. Through the IKKβ of *Xenopus laevis* is highly homologous to *Homo sapiens*’ (Identities = 76%, Positives = 86%, seen in File S1 about the sequence alignment result), the Keap1 binding motif of IKKβ is slightly different between *Xenopus laevis* and *Homo sapiens* (Figure 3). In order to analyze the recognition model of Keap1 and IKKβ in *Homo sapiens*, the homology model of human IKKβ was built depending on the crystal structure of *Xenopus laevis*’ IKKβ (Figure 4B). The combination of high sequence identity and high resolution of the template structure ensure the quality of homology model to be sufficiently good to allow the structural and functional research. The best model, as determined by the lowest value of the Modeler objective function and the least ‘discrete optimized potential energy’ (DOPE) score, was optimized by simulated annealing. The compatibility score resulted from Profile-3D analysis tool in DS 3.0 also proved the reliability of the homology model (File S2). The three dimensional alignment revealed that the homology model did not differ from the template in the peptide backbone (Figure 4C). IKKβ contains a kinase domain (KD), a ubiquitin-like domain (ULD) and an elongated, α-helical scaffold/dimerization domain (SDD) [32] (Figure 4A). The Keap1 binding motif of IKKβ, located in the KD, forms a β-turn region.

### Protein-Protein Docking

**Figure 5:**
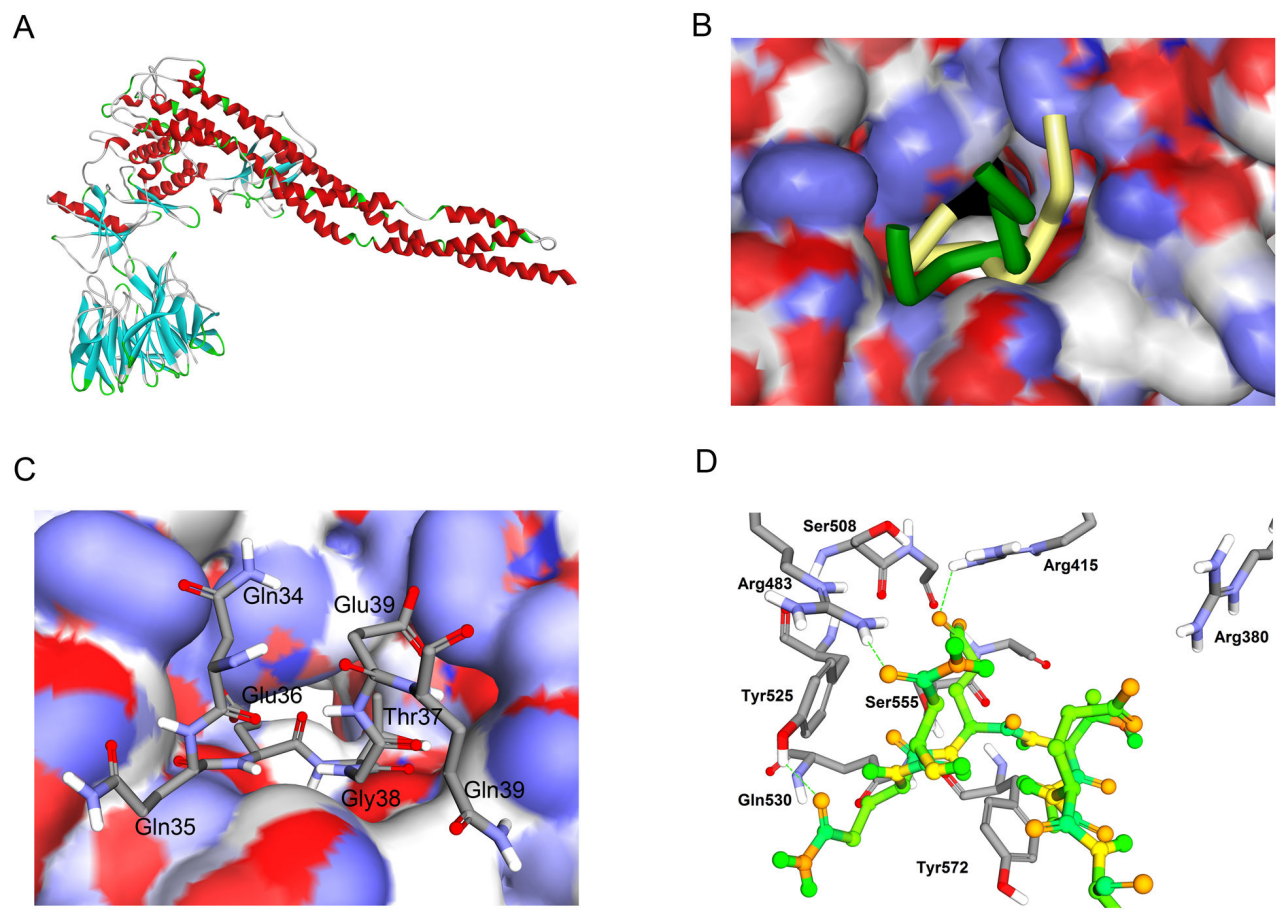
Protein-Protein Docking of IKKβ and Keap1. (A) Complex of IKKβ and Keap1 shown as ribbon diagram. The Keap1 binding motif of IKKβ fits into an identical pocket of Keap1. (B) Superimpose of IKKβ (Dark green) and Nrf2 (yellow, PDB ID: 2FLU) ETGE motif in the Keap1 cavity. IKKβ and Nrf2 occupy almost the same part of Keap1 cavity. However, the structures of ETGE motif show some differences. In the case of IKKβ, the two antiparallel β-strands, forming the turn region, are closer than the Nrf2 ETGE motif. (C) The top view of Keap1-IKKβ complex. The IKKβ ETGE motif is represented in sticks and the surface of Keap1 is colored by partial charge. Both of the two side chain carboxyl groups point to the positively charged surface. (D) Interacting amino acid residues on the IKKβ and Keap1. The interacting region of Keap1 is represented in sticks and the regions of IKKβ are shown in ball and sticks.

The biological activities of many proteins depend on the specific recognition of one or more partner proteins. The most important method for obtaining structural information on protein-protein interactions is protein-protein docking [46-50]. The known binding motifs of IKKβ and Keap1 ensure the accuracy of the docking results. After refining the docking results, 100 poses were kept for further consideration. Energy of RDOCK was used to rank the remaining poses. Top 20 pose were projected to visual analysis. Pose 33, the second top pose, was finally selected for its similar binding location to Keap1-Nrf2 system (Figure 5A). However, the structures of ETGE motif show some differences. In the case of IKKβ, the two antiparallel β-strands, forming the turn region, is closer than the Nrf2 ETGE motif (Figure 5B). The Glu36 and Glu39 of IKKβ possessed electrostatic interaction towards Arg380 and Arg415 of Keap1, similar to the Glu79 and Glu82 of Nrf2 did (Figure 5D). Meanwhile, the side chain of Arg483 of Keap1 which was slightly away from the Glu36 but close to /Asn34, forming a hydrogen bond with the side chain O of Asn34. The Gln35 (side chain O) formed a hydrogen bond with the phenolic hydroxy of Tyr525, which didn’t present in the crystal structure of Keap1-Nrf2 system. In general, the interaction model of Keap1-IKKβ, obtained from protein-protein docking protocol, was resemble to the Keap1-Nrf2 system. These results are also accordant with the published result [15].

### Molecular Dynamic Simulation

**Figure 6:**
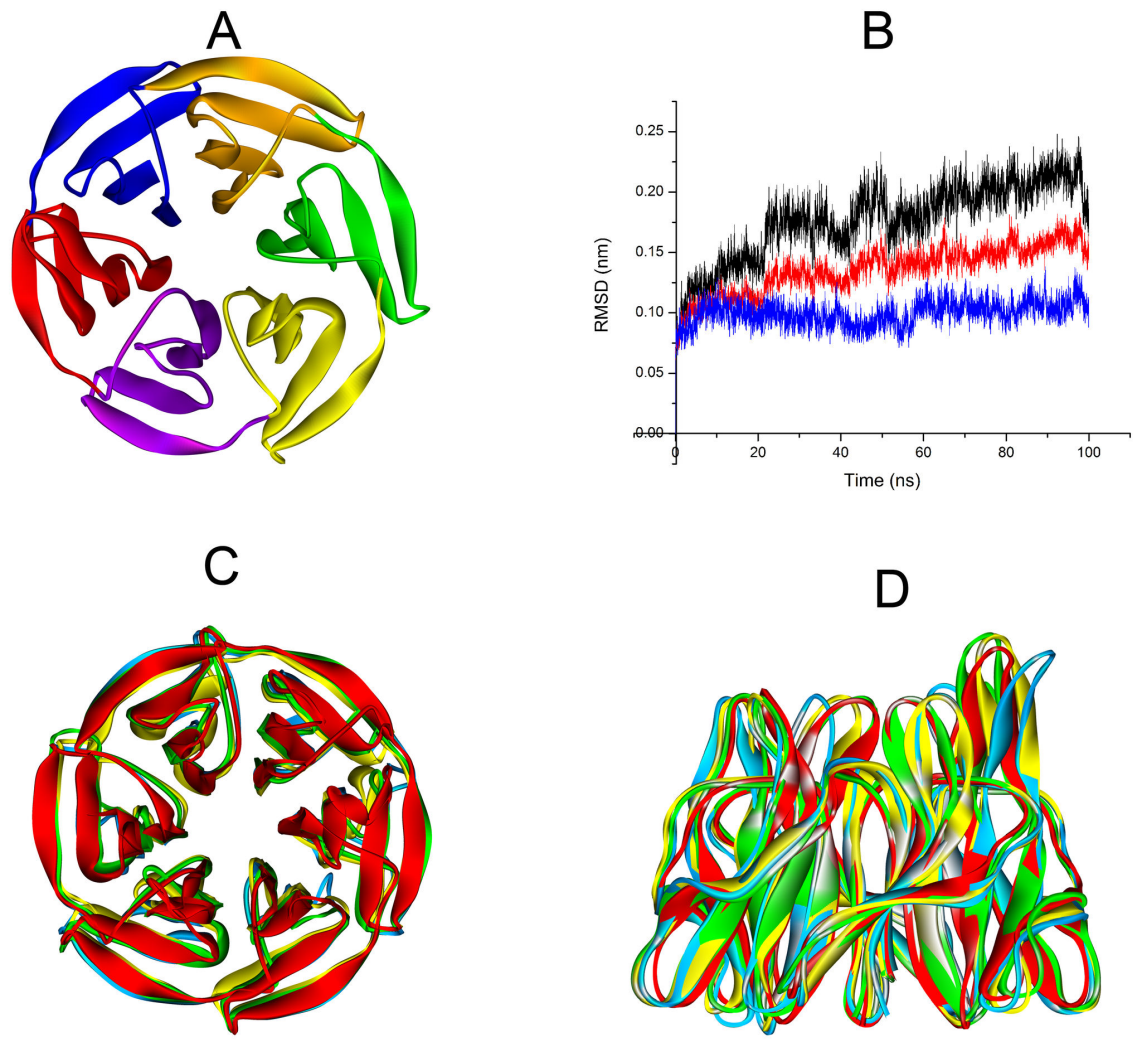
MD simulation of Keap1 DC domain. (A) The six kelch repeats that comprise the DGR domain of Keap1 form a highly symmetric, 6-bladed-propeller structure (Blade I: residue 598-609 and 327-358, blue; Blade II: residue 359-409, red; Blade III: residue 410-456, purple; Blade IV: residue 457-503, yellow; Blade V: 504-550, green; Blade VI: residue 551-597, orange PDB code: 2FLU). (B) Analysis of Root Mean Square Deviation of backbone atoms during molecular dynamics simulation (red for the first run, blue for the second run and black for the third run). (C) & (D) Representative structures of triplicate MD simulations (coloured as azure, yellow and green) superimposed to the starting structure (red). Though the whole structure of Keap1 DC domain is stable, all six blades of the β-propeller tend to be more open and loose.

The six Kelch repeats that comprise the DGR domain of Keap1 form a highly symmetric, 6-bladed-propeller structure [51] (Blade I: residue 598-609 and 327-358, blue; Blade II: residue 359-409, red; Blade III: residue 410-456, purple; Blade IV: residue 457-503, yellow; Blade V: 504-550, green; Blade VI: residue 551-597, orange. PDB code: 2FLU Figure 6A). In order to evaluate the structure variability of Keap1 DC domain, 100 ns MD simulation was conducted under the NPT ensemble for three times. Figure 6B shows the Root Mean Square Deviation (RMSD) of backbone atoms of Keap1 DC domain. The RMSD value is under 0.25 nm through the whole MD process of triplicate simulations, which indicates good protein stability over the course of the simulation. The representative structures obtained from the cluster analysis were saved to compare the original structure with the MD equilibrium structure. The cluster size can be found in File S3. As shown in Figure 6C& D, though the whole structure of Keap1 DC domain is stable, all six blades of the β-propeller tend to be more open and looser after MD simulation. It indicated that the cavity of Keap1 is contracted by induced fit interaction between Keap1 and the substrate. Part of that was because the electrostatic repulsion between the positively charged arginines in the cavity of Keap1.

**Figure 7:**
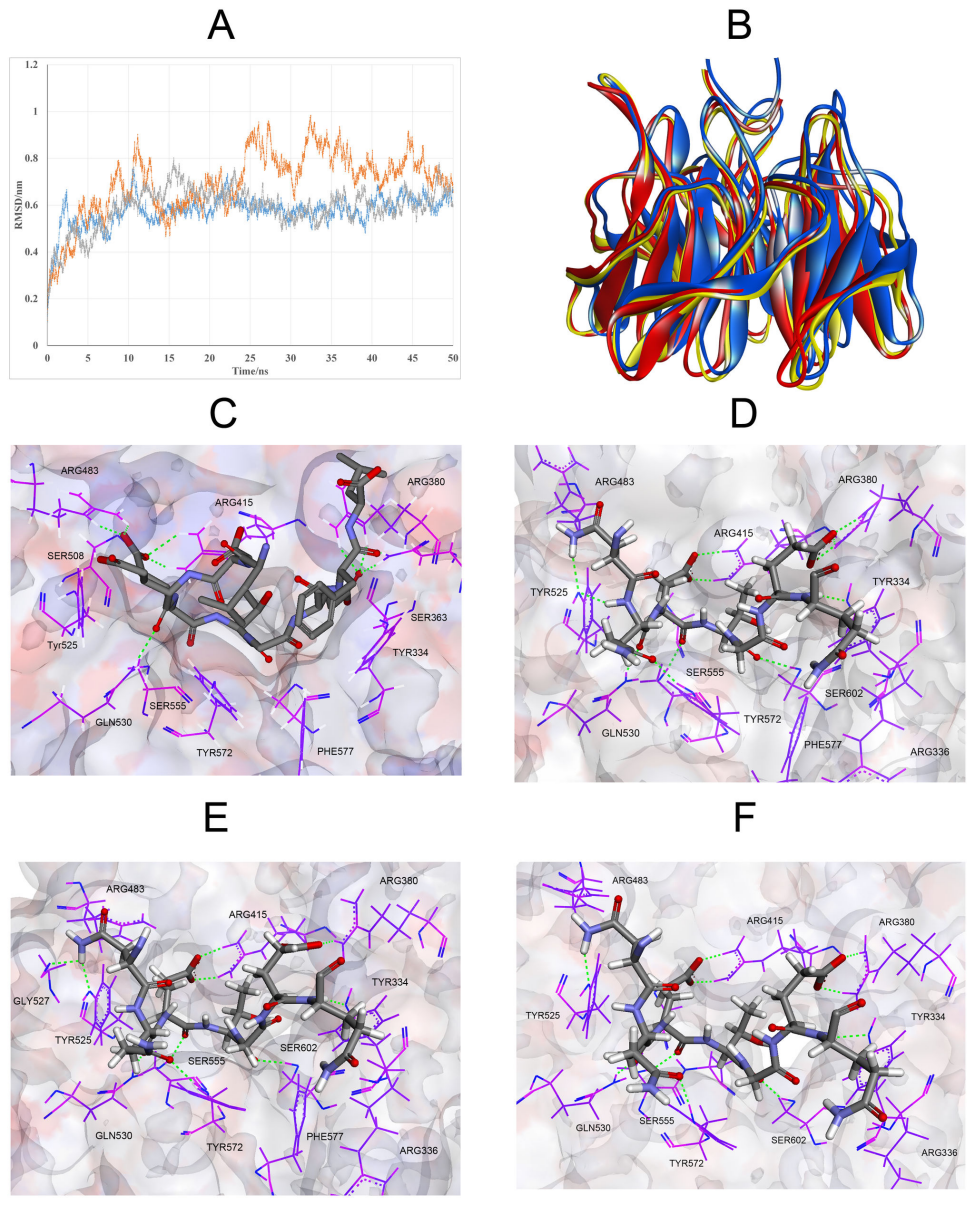
MD simulation of IKKβ-Keap1 complex. (A) RMSD value of backbone atoms respect to the starting structure (yellow for the first run, gray for the second run and blue for the third run). (B) Superimposition of three representative structures of triplicate MD simulations (coloured as red, yellow and blue). Only the ETGE motif was shown in the figure. The Keap1 DC domain is stable during the simulation. (C) Interaction model of Keap1-Nrf2. (D) (E) & (F) Binding model of three representative structures of triplicate MD simulations. The residues of Keap1 are represented as sticks and the residues of ETGE motif are represented as ball and sticks. Hydrogen bonds are shown as green dashed line.

We have also simulated Keap1-IKKβ complex from the protein-protein docking results for 50 ns. Total energy and potential energy of the system are constant during the MD simulation (File S4). RMSD values of backbone atoms respecting to the starting structure (Figure 7A) indicate the system tends to be stable after 20 ns, through in the first run, the RMSD of the backbone have some cyclical swings in the simulation. The central structure of the most dominant clusters for each of the simulations was saved to analyze the interaction of Keap1-IKKβ. All of the most representative structures have a similar binding pattern (Figure 7C). Compared to the starting structure, the binding of Keap1-IKKβ is much tighter in all of the three replicate simulations. As shown in Figure 7D, E & F, the Glu36 and Glu39 of IKKβ form salt bridges and multiple hydrogen bonds to Arg380 and Arg415 of Keap1, respectively. Meanwhile, the carboxylic group is closer to the guanidine group to improve the binding affinity. The Gln35 (backbone O) of IKKβ possesses hydrogen bond to the Gln530 (side chain NH), similarly to the Glu78 (backbone O), which is also conserved in the three replicates. Thr37 (backbone O) of IKKβ, as the same as Thr80 of Nrf2, forms hydrogen bond with the hydroxyl group of Ser602 of Keap1. However, different from the Nrf2-Keap1 system, three key tyrosines of Keap1 play a key role in the recognition of IKKβ. Asn34 (side chain NH), Gln35 (side chain O) and Gln40 (backbone O) form hydrogen bonds with Tyr525, Tyr574 and Tyr334 of Keap1, respectively. Besides, the Gln35 (backbone NH) also form hydrogen bond with Tyr525 in one of the triplicate simulations (Figure 7D). In another representative structure, Asn34 (side chain NH) form multiple hydrogen bonds with Tyr525 (side chain O) and Gly527 (backbone O) (Figure 7E). These results indicate that Tyr525 may be the key residue to recognize IKKβ. Trp, the most important residue in terms of both conservation and free energy change in Ala scanning is remarkable in many PPIs [52]. It is not amazing that the tyrosine is exploited by Keap1 to recognize ubiquitinaion substrate. While in the case of Nrf2-Keap1, the Asn34 and Gln35 of IKKβ change to the Asp77 and Glu78. Considering the cavity is surrounded by the basic amino acid residues, especially arginines, both the carboxyl group and the phenol group could be negatively charged. Thus, not only the formation of hydrogen bonds could be disrupted, but the electrostatic repulsion may displace the responding residues. Nevertheless, the interaction of Keap1 and Nrf2 highly rely on the electrostatic interactions between glutamic acid and arginine. As show in Figure 7C, the Glu79 of Nrf2 forms salt bridge and multiple hydrogen bonds to Arg483 and Arg415 simultaneously, while the Glu82 of Nrf2 can form hydrogen bond to Arg380 and Asn382. Besides, the salt bridge between Glu82 and Arg380 also exists. These differences described above indicate that three tyrosines, namely Tyr525, Tyr574 and Tyr334, are the key point to selectively bind IKKβ, while three arginines play an important role in recognizing Nrf2. Considering the extra interactions with tyrosines, the binding affinity of Keap1 and IKKβ may be higher than the Keap1-Nrf2 system. These results also reveal that selective PPI inhibitors of Keap1-Nrf2 should focus on the key arginines while selective PPI inhibitors of Keap1-IKKβ should pay attention to the key tyrosines.

### Virtual Alanine Mutation of binding residues

**Figure 8:**
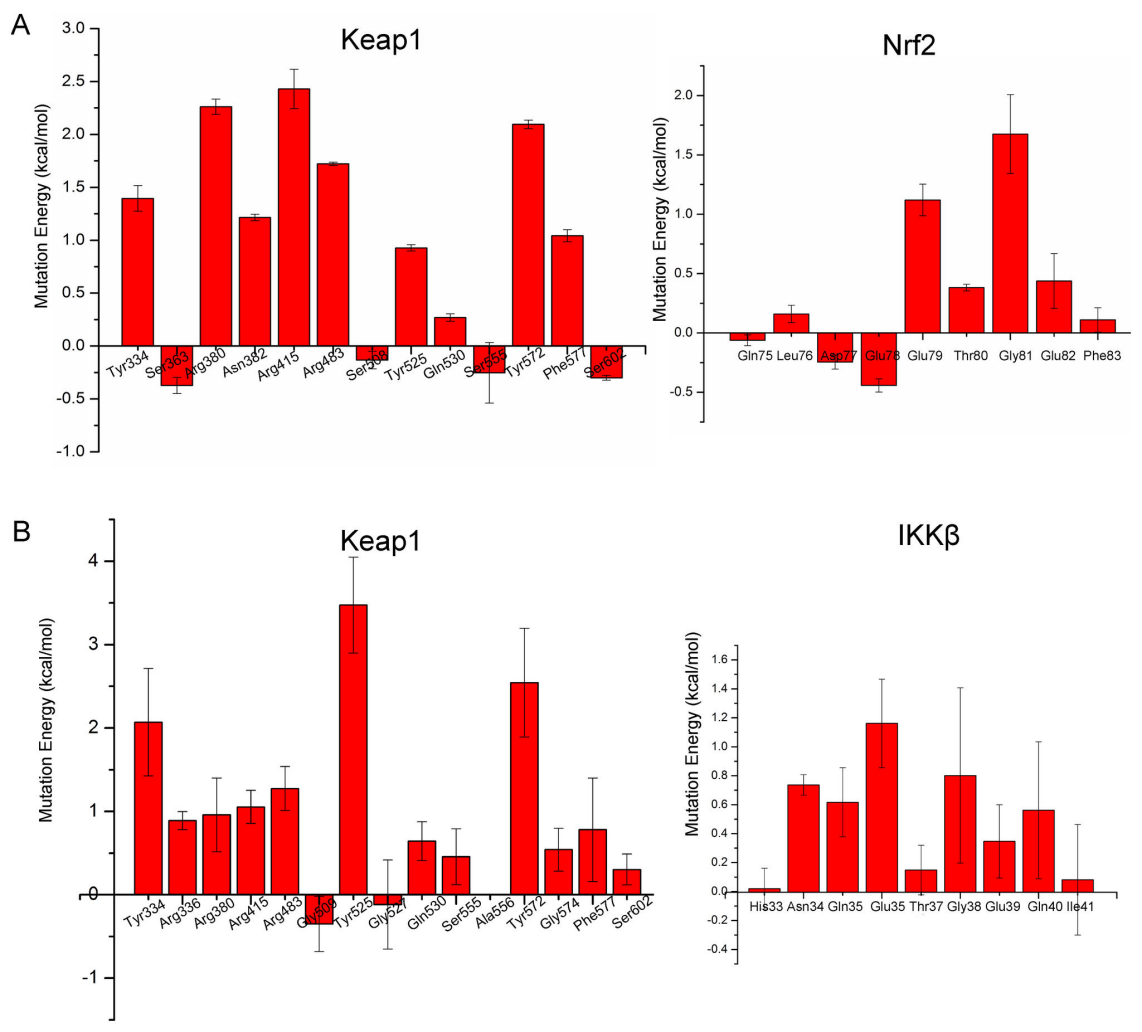
Virtual Alanine Mutation of binding residues. The Y axis is the weighted mutation energy (unit: kcal/mol) and the X axis is the name of mutated amino acid. (A) Mean values of Virtual Alanine Mutation of Nrf2-Keap1 complex with standard deviation. (B) Mean values of Virtual Alanine Mutation of Keap1-IKKβ complex obtained from triplicate MD simulations with standard deviation.

Virtual Alanine Mutation Scanning (VAMS) on the binding residues was performed to evaluate the energetic effect of each residue on the binding affinity. The mean values of Virtual Alanine Mutation of Keap1-IKKβ complex obtained from triplicate MD simulations with standard deviation were shown in Figure 8B. In the case of Keap1-IKKβ, three tyrosines of Keap1, namely Tyr525, Tyr574 and Tyr334, are responsible for the highest energy effect for binding which is consistent with analysis of binding model described above. VAMS of IKKβ show that besides the Glu36 and Glu39 which take part in the electrostatic interactions with arginines, Asn34 and Gln35 which can form hydrogen bonds with tyrosines are also important for binding. The mutation of Gln40 in the VAMS procedure also destabilize the binding remarkably indicating the side chain interaction with Arg336 of Keap1 may contribute to the binding affinity. It is also consistent with the result that the alanine mutation of Arg336 also can destabilize the binding. In both of Nrf2-Keap1 and IKKβ-Keap1 systems, the alanine mutation of glycine in the ETGE motif could destabilize the complex manifesting the intolerance of a methyl substitution. In consistent with experimental alanine mutations [53], the VAMS also indicate that the Glu79 and Glu82 of Nrf2 have a stronger driving force in substrate recognition. VAMS of Keap1 generated similar results. Three arginines seemed to be important for recognition of Nrf2. Besides, Tyr572 also contributes to binding through the van der Waals interaction.

Taken together, the MD simulations and Virtual Alanine Mutation Scanning combine to indicate the different substrate recognition mechanism of Keap1. In the case of Nrf2-Keap1 system, three arginines of Keap1 play an important role in recognizing the Nrf2 through interacting with the key glutamic acids. Although the arginine-glutamic acid interactions also exist in the IKKβ-Keap1 system, the tyrosine, especially Tyr525, participated interactions dominate the recognition of IKKβ. These differences may be utilized by the organism to fine tune the Keap1-Cul3 E3 ligase complex to modulate Keap1-Nrf2-ARE and IKKβ-Nf-κB pathway simultaneously.

## Discussion

Degradation by ubiquitination is a common strategy taken by the key modulators responsible for stress responses [1,54,55]. The selective ubiquitination of the substrate controlled by the E3 ligase is the key step for the repression regulation [56]. In the case of Keap1-Cul3 E3 ligase complex, the PPI with Keap1 determine which protein should be ubiquitination and further degraded by 26S proteasome which can cause dramatic changes of downstream signaling.

IKKβ and Nrf2 are the primary substrates of Keap1. IKKβ can activate the transcription factor NF-κB that is directly involved in inflammation and tumor progression. Furthermore, IKKβ has been shown to promote tumorigenicity through phosphorylation-mediated inhibition of tumor suppressors and thus is considered as an oncogenic kinase [23,57,58]. While another Keap1 substrate, Nrf2, has been known as an attractive target for chemprevention of cancer [59]. Considering Keap1-Nrf2-ARE and IKKβ-NF-κB pathways play contrary roles in the pathological processes of inflammation and cancer, the interference of NF-κB signaling and Nrf2-ARE pathway is vital to maintain the balance. Some preliminary findings about crosstalk between the NF-κB signaling and Nrf2-ARE pathway have been reported. It has been found that NF-κB signaling can inhibit Nrf2-ARE pathway through the interaction of p65 and Keap1 [21]. In another study, p65 is involved in down-regulation of expression of anti-oxidative genes through the deprivation of CBP from Nrf2 and promotion of the recruitment of HDAC3 to ARE [60]. These results show that NF-κB can antagonize Nrf2-ARE pathway through the mediation of p65.

Herein, depending on the recognition mechanism of Keap1 and IKKβ, we postulate a new regulatory mechanism between Keap1-Nrf2-ARE and IKKβ-NF-κB pathway mediated by Keap1. The results described above reveal that Nrf2 and IKKβ are recognized by Keap1 through similar but not identical mechanism, indicating the competitive binding of IKKβ and Nrf2. In basal conditions, overexpression of IKKβ may result in the derepression of Nrf2 via competive binding Keap1. It is consistent with published result that p65 and most of its upstream molecules repress ARE-Luc reporter and NQO1-Luc reporter activity with varying degrees, while IKKβ is an exception [21]. In induced conditions, electrophilic modification of specific Keap1 cysteines induces conformational changes in Keap1, which could lead to the derepression of Nrf2 and IKKβ simultaneously. It can explain why some inflammation stimuli, such as ROS and electrophilic reagents, can activate both NF-κB signaling and Nrf2-ARE pathway. The activation of Nrf2 will clean up the inflammation stimuli and restore the redox balance, which can in turn recover the function of Keap1. Finally, the reactivation of Keap1 can turn off the Keap1-Nrf2-ARE and IKKβ-Nf-κB pathways. Thus, it can be easily understood that the deficient of Nrf2 could lead to greater activation of NF-κB in response to inflammation stimuli [61]. Furthermore, mass spectrometry of Keap1 protein treated with electrophilic reagents in vitro demonstrates that different electrophilic reagents give rise to different patterns of cysteine modification of Keap1 [56,62-66]. Selective cysteine modification could cause the specific conformation changes of Keap1 which may distinguish the specific substrate to bind. Thus, it’s possible that specific electrophilic reagents may selectively activate Nrf2 or IKKβ which can also be used in the rational design of selective small compounds to active Nrf2. Besides, multiple component disruption of Keap1-Cul3-RBX1 complex could be a novel mechanism of NF-κB activation [67].

Activation of ARE system by negatively controlling the Keap1 protein holds a great promise for the development of novel class of antiinflammatory and anticancer agents [68]. However, uncontrolled activation of Nrf2 through inhibition of Keap1 could cause the upregulation of tumorigenicity kinase, IKKβ. Thus, pharmacological agents designed for inhibition of Keap1 should be fine-tuned to selectively activate Nrf2.

In summary, the intermolecular recognition mechanism of Keap1 and IKKβ was investigated through protein-protein docking, MD simulations and virtual alanine mutation. Key tyrosines were firstly found to be important for the PPI of Keap1-IKKβ. The presented PPI model of IKKβ-Keap1 with structural information will aid in the rational design of small molecular PPI inhibitors to manipulate the transactivation activity of Nrf2 for therapeutic treatments of stress-related diseases. The crosstalk between Keap1-Nrf2-ARE and IKKβ-Nf-κB pathways mediated by Keap1 proposed in this paper also provides valuable insights into the regulatory mechanisms of cellular redox homeostasis.

## Supporting Information

File S1 **Sequence alignment of *Homo sapiens* IKKβ and *Xenopus laevis* IKKβ (PDB code: 3QA8).**
(DOCX)

File S2 **Profile-3D analysis of *Homo sapiens* IKKβ.** The total Verify Score is 209.87 and verify scores of most residues are greater than 0. Even these residues bearing verify score less than 0 are far away from the binding site.
(DOCX)

File S3 **Total energy and potential energy during the MD simulations of Keap1-IKKβ.** As shown in figure, total energy and potential energy of systems are constant during the MD simulation.
(XLSX)

File S4 **Detailed information of clustering analysis.**
(DOCX)

File S5 **The mdp files used in the MD simulations.**
(ZIP)

File S6 **The related structures, including homology model of human IKKβ, the protein-protein results and structures used for alanine scanning calculations obtained from the triplicate MD simulations, used in the paper.**
(ZIP)
