# Supplementary material for: Insight into the Intermolecular Recognition Mechanism between Keap1 and IKKβ Combining Homology Modelling, Protein-Protein Docking, Molecular Dynamics Simulations and Virtual Alanine Mutation
Source: PLoS One. 2013 Sep 16;8(9):e75076. doi: 10.1371/journal.pone.0075076 (PMC3774807; doi:10.1371/journal.pone.0075076)
Supplement: File S1 — Sequence alignment of Homo sapiens IKKβ and Xenopus laevis IKKβ (PDB code: 3QA8). (DOCX) [file pone.0075076.s001.docx]

Supporting Information S1


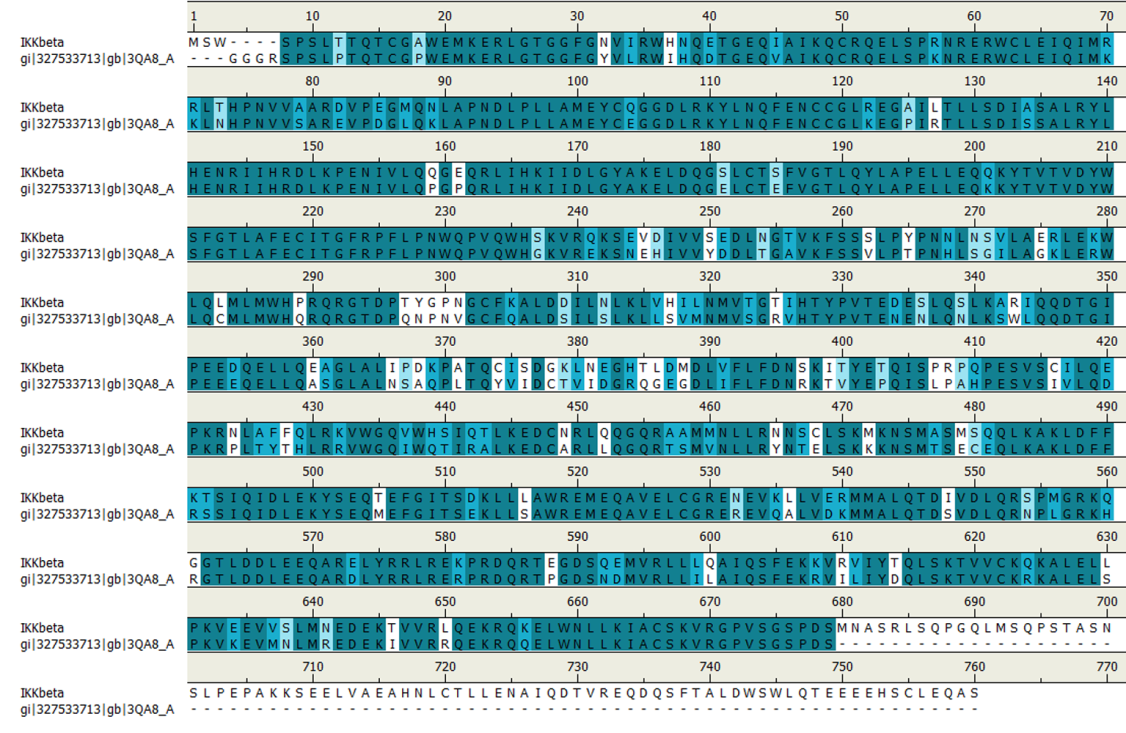


S1. Sequence alignment of *Homo sapiens* IKKβ and *Xenopus laevis* IKKβ (PDB code:3QA8)
