# Supplementary material for: Insight into the Intermolecular Recognition Mechanism between Keap1 and IKKβ Combining Homology Modelling, Protein-Protein Docking, Molecular Dynamics Simulations and Virtual Alanine Mutation
Source: PLoS One. 2013 Sep 16;8(9):e75076. doi: 10.1371/journal.pone.0075076 (PMC3774807; doi:10.1371/journal.pone.0075076)
Supplement: File S2 — Profile-3D analysis of Homo sapiens IKKβ. The total Verify Score is 209.87 and verify scores of most residues are greater than 0. Even these residues bearing verify score less than 0 are far away from the binding site. (DOCX) [file pone.0075076.s002.docx]

Supporting Information S2





S2. Profile-3D analysis of *Homo sapiens* IKKβ. The total Verify Score is 209.87 and verify scores of most residues are greater than 0. Even these residues bearing verify score less than 0 are far away from the binding site.
