# Supplementary material for: Insight into the Intermolecular Recognition Mechanism between Keap1 and IKKβ Combining Homology Modelling, Protein-Protein Docking, Molecular Dynamics Simulations and Virtual Alanine Mutation
Source: PLoS One. 2013 Sep 16;8(9):e75076. doi: 10.1371/journal.pone.0075076 (PMC3774807; doi:10.1371/journal.pone.0075076)
Supplement: File S4 — Detailed information of clustering analysis. (DOCX) [file pone.0075076.s004.docx]

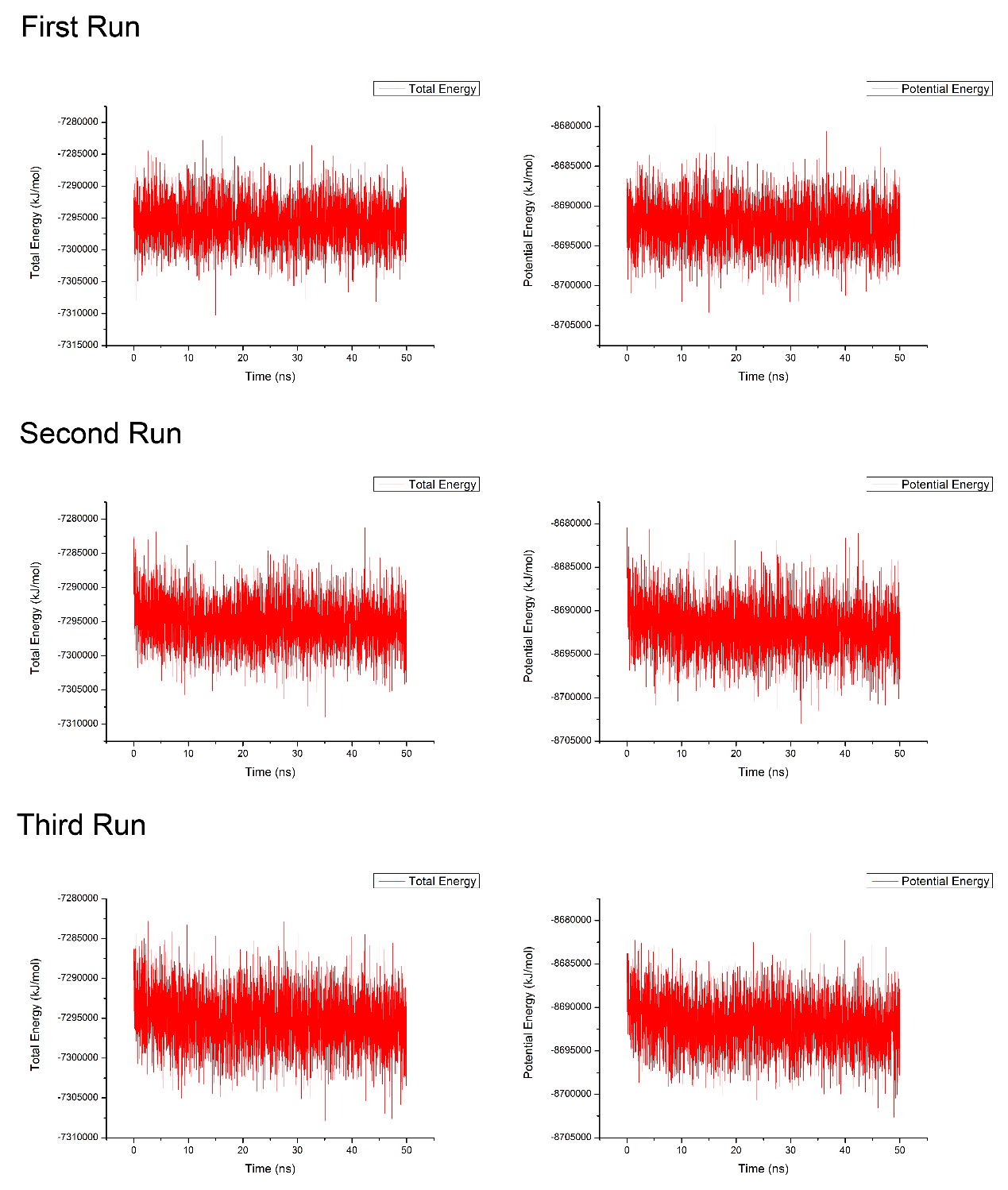


S3. Total energy and potential energy during the MD simulations of Keap1-IKKβ. As shown in figure, total energy and potential energy of systems are constant during the MD simulation
